# Targeting enabled homolog with daunorubicin inhibits ERK1/2/c‐Fos pathway and suppresses hepatocellular carcinoma progression

**DOI:** 10.1002/ctm2.70366

**Published:** 2025-06-09

**Authors:** Zhi‐Mei Li, Guan Liu, Qing‐Qing Liu, Wei‐Ming You, Ming‐Gao Zhao

**Affiliations:** ^1^ Institute of Medical Research, Northwestern Polytechnical University Xi'an Shaanxi China; ^2^ Department of Pharmacy Tangdu Hospital, Fourth Military Medical University Xi'an Shaanxi China; ^3^ Department of infectious Diseases Department of Tumor and Immunology in Precision Medical Institute and Western China Science and Technology Innovation Port The Second Affiliated Hospital of Xi'an Jiaotong University Xi'an Shaanxi China

1

Dear Editor

The enabled/vasodilator stimulated phosphoprotein family (Ena/VASP) of actin regulatory proteins are critical players in maintenance of cell shape, motility, regulation of molecular interactions, and contribute to hepatocellular carcinoma (HCC) development,^1^ rendering them ideal candidate targets for therapy, whereas understanding their regulatory mechanisms remains an unmet challenge and no sufficiently potent compounds to interfere with Ena/VASP in HCC have been reported.^2^, ^3^ In this context, we identified enabled homolog (ENAH), a member of Ena/VASP, as a promising target for pharmaceutical intervention derived from multi‐omics analysis. Using biochemical and molecular biology assays, for the first time we report daunorubicin, a known drug used to treat acute myeloid leukaemia, as an ENAH inhibitor. This study aims to uncover the underlying mechanism and investigate the therapeutic potential of daunorubicin in preclinical HCC models.

We collected resected primary tumour samples and normal paired adjacent tissues (NATs) from 20 early‐stage HCC patients without radiotherapy or prior chemotherapy (Table S1). Four pairs of qualified samples were selected for proteomic and transcriptomic analyses. Using isobaric standard mass tag labelling, 371 differentially expressed proteins (DEPs) were identified in tumours compared to NATs (Figure S1A and C and Table S2). mRNA sequencing (mRNA‐seq) profiled 24 381 genes, among which 1888 and 1924 protein‐coding genes were significantly down‐ and upregulated, respectively, in HCCs (Figure S1B and D and Table S3). DEPs featured biological activities of metabolic processes, focal adhesion, and actin binding function. Compared with proteomic data, mRNA‐seq profile additionally exhibited functional enrichment with GTPase activity, MAPK cascade, ERK1/ERK2, and other signalling pathways in HCC (Figure S1E–H and Table S4).

Among 3812 dysregulated protein‐coding genes, 56 showed concordances between mRNA and protein abundance (Figure 1A). Signatures specific to the amino acid metabolic process and ERK1/2 regulation were significantly enriched in the tumours (Figure S2A). In particular, novel molecules such as ENAH, HOGA1, and FGFR2, were related to focal adhesion, cytoskeleton organisation, and actin filament process, which are implicated in tumourigenesis and metastasis. Using correlation analysis, 32 proteins were identified as representative signatures associated with HCC (Figure S2B), of which 20 were significantly dysregulated in TCGA dataset (Figure S2C). Among them (highlighted in Figure 1B), seven molecules, especially TPX2, CDCA3, and ENAH, displayed very high risk scores for the mortality prognosis of HCC. Here, we focused on ENAH, which exhibited upregulated mRNA levels associated with higher stage/pathological grade/poor clinical prognosis of patients and has been reported to be a critical effector mediating cellular cytoskeletal motility related to tumour development^4^ (Figure S2D–G, Tables S5 and S6). IHC staining confirmed ENAH overexpression in HCC and its usefulness as a feature marker (Figure S2H and I and Table S7). In addition, ENAH showed higher abundance in most tumours, such as lung adenocarcinoma and cholangiocarcinoma, than in normal tissues (Figure S2J). The Human Pathology Atlas data indicated that elevated ENAH levels were also significantly correlated with overall survival (OS) in urothelial cancer, renal cancer, and breast cancer.

**FIGURE 1:**
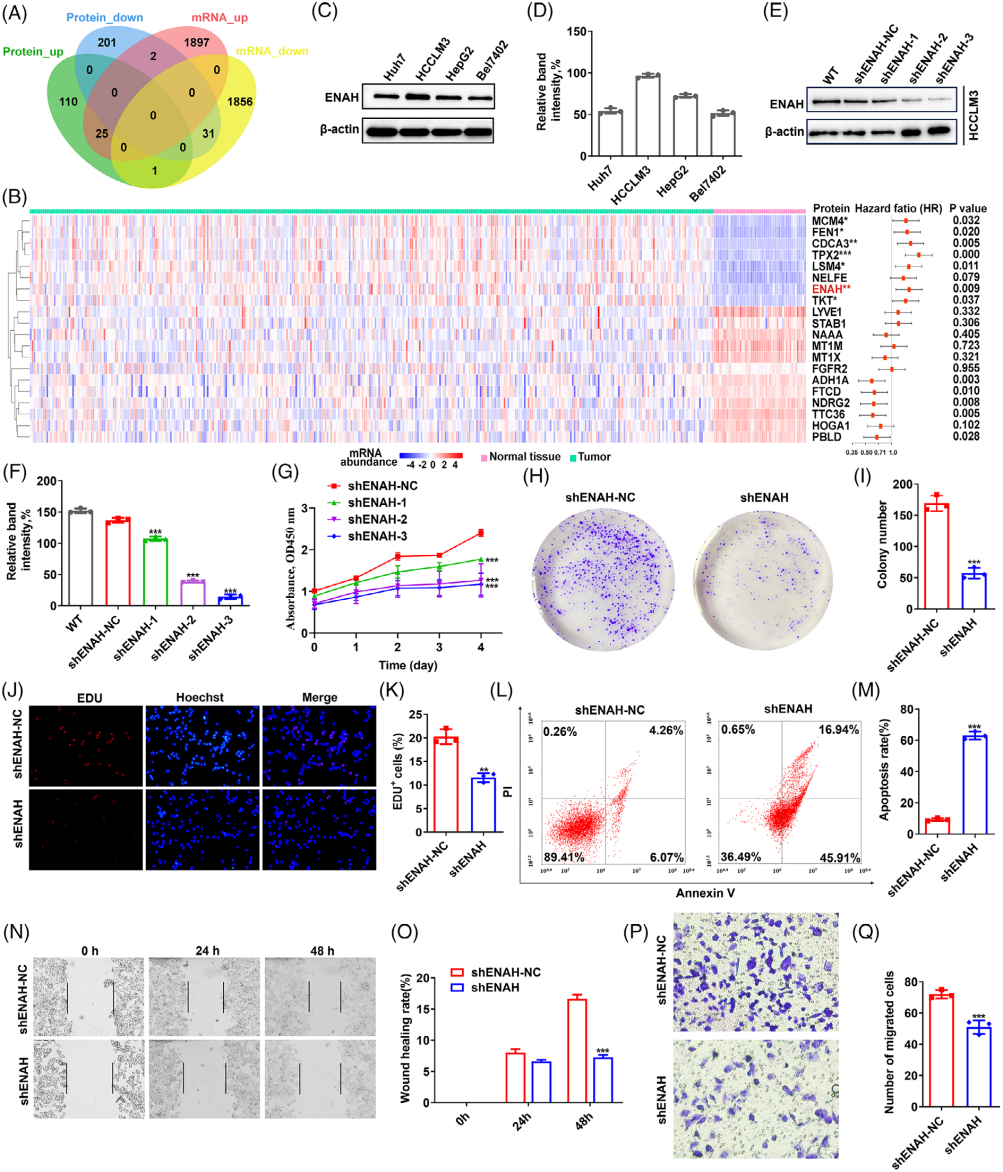
Identification of ENAH as a potential therapeutic target for HCC. (A) Venn diagram illustrating the overlap of differentially expressed proteins and genes among our proteomic and transcriptomic profiles of HCC. (B) Potential signature proteins for HCC development. Left, relative abundance of the proteins in TCGA cohort. Right, hazard ratios (HR) of each protein shown as ‘HR (95% confidence interval)’. The signature proteins are highlighted by asterisks. (C) ENAH expression level in four HCC cancer cell lines was determined using western blot analysis. (D) The quantification data for the band intensity of ENAH immunoblots in (C). (E) Establishment of the ENAH knockdown‐HCCLM3 cell line, verified by immunoblot. (F) The quantification data for the band intensity of ENAH immunoblots in (E). (G) Effect of ENAH knockdown on cell proliferation, evaluated using the CCK‐8 assay. (H) ENAH knockdown promoted the colony formation abilities of HCCLM3 cells. (I) Quantification data for (H). (J) ENAH knockdown increased the EDU‐positive staining cells following the EDU incorporation assay in HCCLM3 cells. (K) Quantification data for (J). (L) Representative flow cytometry apoptosis graphs of control and ENAH knockdown in HCCLM3 cells. (M) Quantification of apoptosis, determined with Annexin V and PI for (L). (N) ENAH knockdown increased the wound healing ability of HCCLM3 cells. (O) Quantification data for (N). (P) The transwell assay for HCCLM3 upon ENAH knockdown. (Q) Quantification data for (P). Quantified results for all the immunoblots normalised to the β‐actin signal compared to reciprocal control. shRNA indicated shENAH‐3. Significance levels are indicated as ^*^ *p*<.05; ^**^ *p*<.01; ^***^ *p*<.001.

To confirm the role of ENAH in HCC progression, we knocked down ENAH in HCCLM3 cells that exhibited the highest expression level of ENAH compared to other human HCC cell lines (Figure 1C–F). We found that ENAH knockdown suppressed cell proliferation, migration and triggered their apoptosis (Figure 1G–Q). Thus, therapies targeting ENAH, such as small molecules, may be beneficial for HCC treatment.

Novel ENAH inhibitors were screened from an in‐house library comprising 2040 compounds using structure‐based virtual ligand screening. Fourteen compounds with prominent docking CDOCKER energies capable of binding ENAH were selected for further investigation (Figures 2A and S3A and Table S8). Four HCC cell lines (HCCLM3, HepG2, Huh7 and Bel7402) with different ENAH expression levels were selected as test models (Figure 1C). As shown in Figure 2B and Table S9, dose‐response curves demonstrated a progressive sensitivity to compound **11**: Bel7402 (low ENAH, IC50 = 41.20 ± 1.61 µM), Huh7 (intermediate ENAH, IC50 = 28.93 ± 1.46 µM), HepG2 (intermediate ENAH, IC50 = 21.15 ± 1.36 µM) and HCCLM3 (high ENAH, IC50 = 7.42 ± 0.06 µM). The sensitivity was correlated with ENAH expression levels in the four cells, indicating that compound **11** (daunorubicin) induced an ENAH‐dependent cancer cell death. The docking models of daunorubicin‐ENAH complexes revealed key interactions, including hydrogen bonds with TRP23, *π*‐*π* stacking with PHE77, and hydrogen bonds with TYR16, LYS21, ARG81 and ALA73 (Figures 2C and S3B–D). In vitro evaluations of daunorubicin showed dose‐dependent inhibition of cell proliferation, apoptosis, migration, and cell cycle analyses showed that daunorubicin increased G2 arrest, and similar effects were observed in ENAH knockdown cells (Figure 2D–P). The interaction between ENAH and daunorubicin was confirmed by the cellular thermal shift assay (CETSA) (Figure 2Q and R). More importantly, ENAH knockdown decreased the inhibitory effect of daunorubicin on cell proliferation (IC_50 _= 34.60 ± 1.83 µM) (Figure 2S). These findings provide powerful evidence that daunorubicin directly interacts with ENAH and inhibits malignant phenotypes of HCCLM3 cells.

**FIGURE 2:**
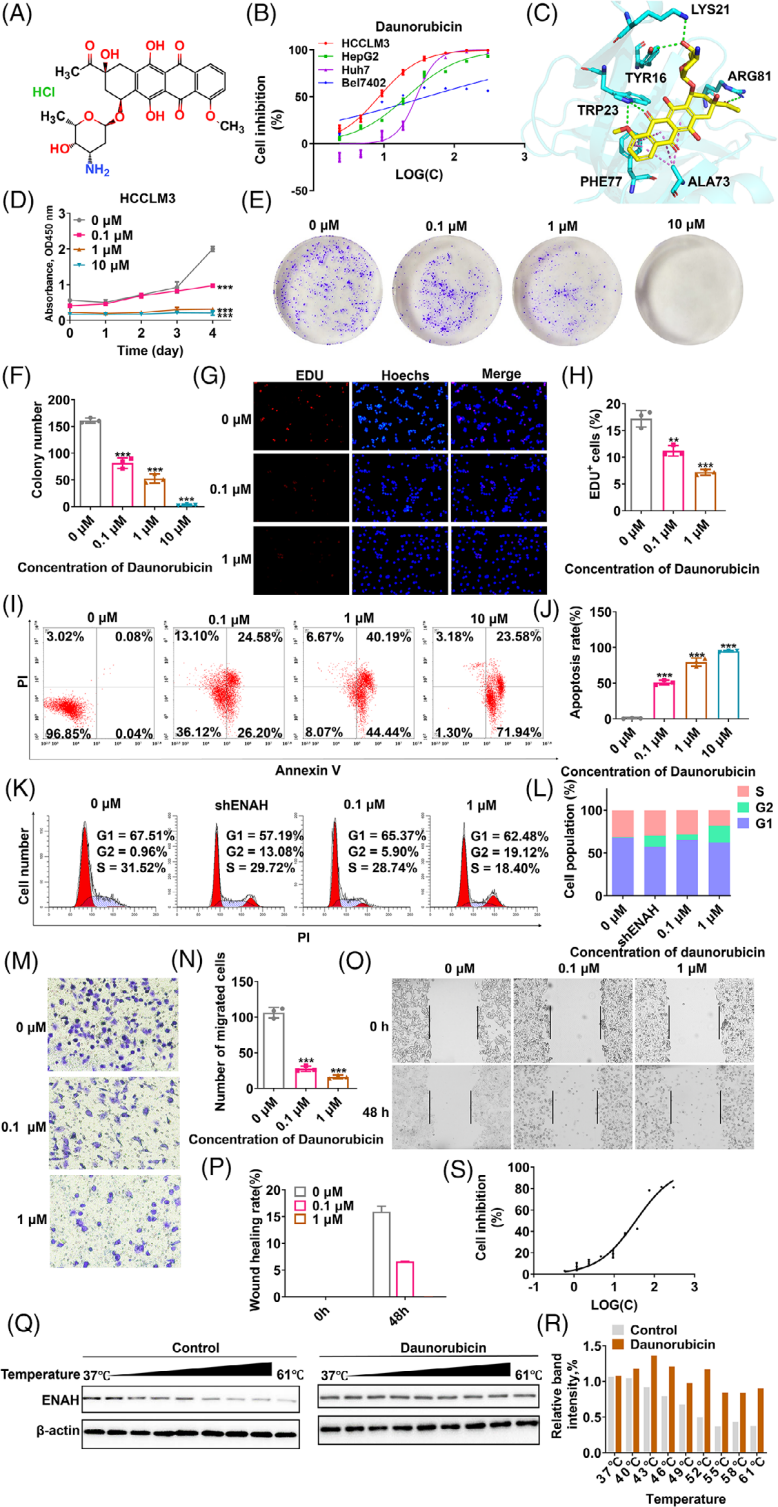
Daunorubicin is a potent ENAH blocker that can inhibit HCC progression. (A) Structure of daunorubicin. (B) Dose‐response curves of HCCLM3, HepG2, Huh7, and Bel7402 cell lines to daunorubicin treatment. (C) Binding conformation and interactions of daunorubicin bound to ENAH generated by molecule docking. (D) HCCLM3 cells were treated with different concentrations of daunorubicin, and cell proliferation was evaluated using the CCK‐8 assay. (E) The colony formation ability of HCCLM3 cells after treatment with daunorubicin. (F) Quantitative analyses for (E). (G) After incubation with different concentrations of daunorubicin, the cell proliferation rate was assessed using an EDU cell proliferation assay kit. (H) The EdU‐positive ratio was quantified to Hoechst‐positive cells. (I) After treatment with daunorubicin at different concentrations for 24 h, HCCLM3 cells were collected for double staining with Annexin V and PI. (J) Quantification of the apoptosis rate for (I). (K) ENAH knockdown or treatment with daunorubicin increased G2 phase arrest. (L) Quantification data for (K). (M) Migration capacities of HCCLM3 cells with/without daunorubicin treatment were compared using transwell assays. (N) Quantification data for (M). (O) Effect of daunorubicin treatment on cell wound healing ability. (P) Quantification data for (O). (Q) CETSA performed in HCCLM3 cells treated with 1 µM daunorubicin or H_2_O control. The stabilisation of daunorubicin on protein ENAH was evaluated by western blot. (R) Quantification of the band intensity (ImageJ) of ENAH and β‐actin immunoblots was shown in (Q). (S) The HCCLM3 cell line upon ENAH knockdown was exposed to varying concentrations of daunorubicin for 24 h to investigate its cytotoxic activity. The quantified results for all the immunoblots were normalised to the β‐actin signal, compared to the reciprocal control. Significance levels are indicated as ^*^ *p*<.05; ^**^ *p* < .01; ^***^ *p* < 0.001.

The ERK1/2/c‐Fos pathway has been described as a linchpin tumourigenic mechanism associated with HCC.^5^ Nevertheless, its diversity and cryptic pharmacologic accessibility pose challenges, new efforts are underway to exploit unrecognised vulnerabilities for novel targeted therapies. Activated p‐ERK1/2/c‐Fos overexpression has been reported to be required for G2 arrest.^6^ Based on previous studies, as expected, ENAH knockdown reduced p‐ERK/c‐Fos expression (Figure 3A and B), and induced G2 arrest (Figure 2K), indicating that ENAH, positioned upstream of the oncogene ERK1/2, can be a new way to regulate this signalling pathway. Bioinformatics analyses showed that 11 839 genes were significantly correlated with ENAH expression, SH3 domain binding, Rho GTPase binding, and cell adhesion molecule binding among the most markedly enriched functions (Figures 3C and S4A and B). ENAH contains the Ena/VASP homology domains EVH1, EVH2, and the central proline‐rich region that interacts with the SH3 and WW domains.^7^ Using SMART tool, we identified Rho GTPase activating proteins (Rho GAPs) with combinations of SH3 and WW domains, including ARHGAP9, ARHGAP12, and ARHGAP27, as potential ENAH interactors. Co‐IP results suggested that ENAH directly binds to ARHGAP9 (Figure 3D), which inactivates Rho GTPases and plays a pivotal role in cell cytoskeleton organisation, migration, proliferation, and cancer formation. Specifically, Rho GTPases display ERK‐activating functions^8^ (Figure S4C). Using bioinformatics analysis, ARHGAP9, which interacted with ENAH, was predicted to affect ERK1/2 signalling networks (Figures 3E and S4D). Comprehensive functional experiments confirmed the critical role of ENAH‐ARHGAP9 interaction in ERK1/2 modulation (Figure 3F and G). Collectively, we reason the mechanism of ENAH as an oncogenic protein capable of activating the ERK1/2/c‐Fos pathway by interacting with ARHGAP9 in HCC cells. The responsiveness of HCCLM3 cells to daunorubicin was attributed to its ability to bind to ENAH and suppress the ERK1/2/c‐Fos signalling pathway (Figure 3H and I).

**FIGURE 3:**
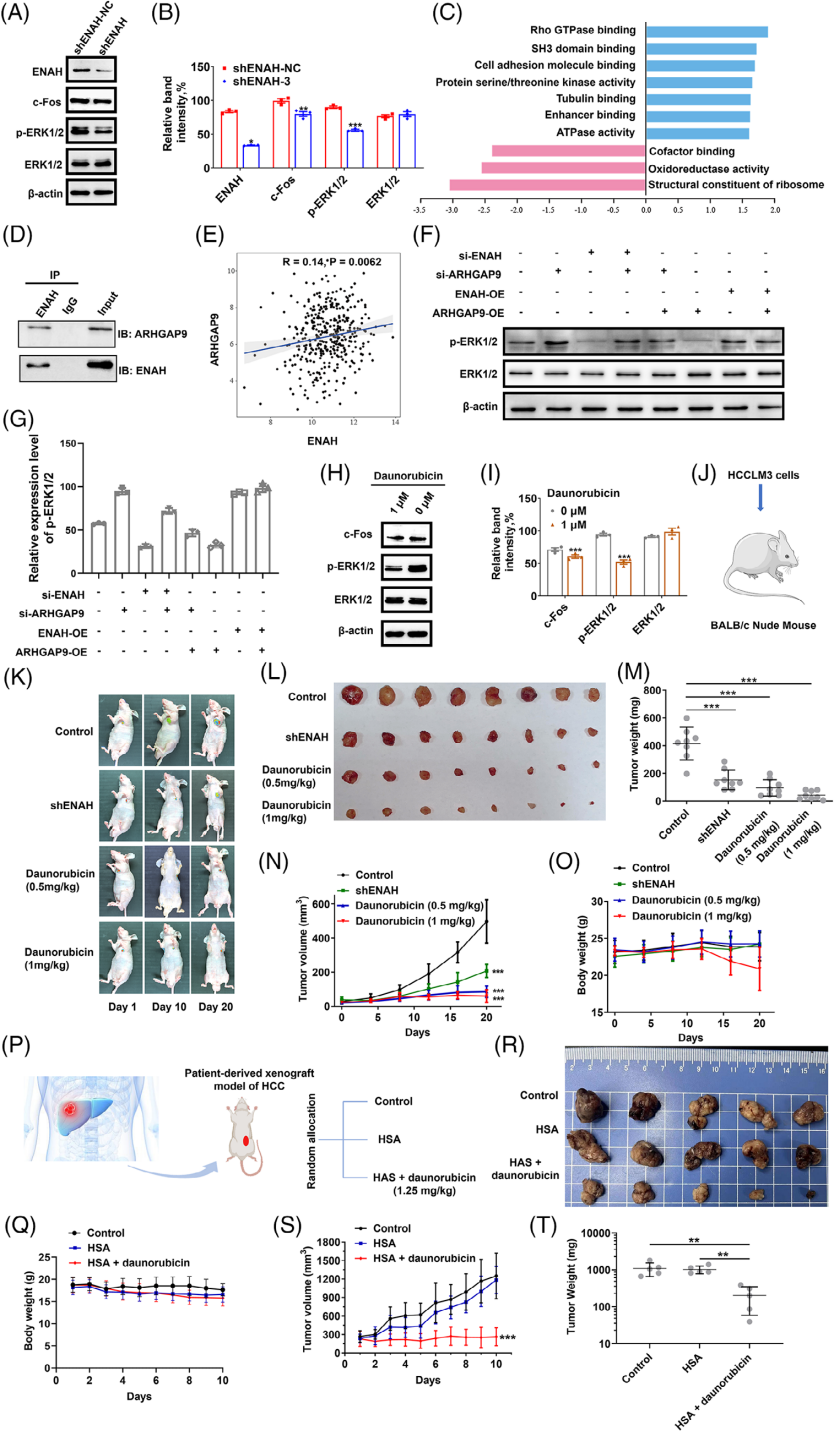
ENAH inhibition with daunorubicin inhibits ERK1/2/c‐Fos pathway and markedly repressed xenograft tumour growth. (A) Immunoblot validation of ERK1/2, p‐ERK1/2, and c‐Fos by ENAH knockdown in HCCLM3 cells. (B) The quantification data for proteins in (A). (C) Functional enrichment analysis of the significant genes in (Figure S4A). (D) Co‐IP assay to determine the protein interaction between ENAH and ARHGAP9. IP: immunoprecipitation, IB: immunoblot. (E) The correlation between the ARHGAP9 level and protein expression of ENAH. The correlation coefficients and *p* values were calculated using the Spearman correlation method. (F) Comprehensive functional experiments (gene knockdown/overexpression) showed that ENAH‐ARHGAP9 interaction critically drives ERK1/2 activation. (G) The quantification data for p‐ERK1/2 in (F). (H) Immunoblot validation of ERK1/2, p‐ERK1/2, and c‐Fos by daunorubicin treatment in HCCLM3 cells. (I) The quantification data for proteins in (H). (J) Schematic illustration of an in vivo therapeutic experiment (CDX model) performed to evaluate the anti‐tumour effect of daunorubicin. (K) Representative in vivo bioluminescence images of mice at the indicated days. (L–O) Representative xenograft tumour images (L), tumour weight (M), quantification data for tumour volumes (N), and mice body weights (O) are shown for the indicated mice groups. (P) Schematic diagram of PDX model construction. (Q) Body weight of mice in each group during the indicated treatments. (R, S) Photographs of tumour (R) and tumour growth curve (S) of mice in each group. (T) Tumour weight of mice in each group after the indicated treatments. Significance levels are indicated as ^*^ *p* < .05; ^**^ *p* < .01; ^***^ *p* < .001.

In vivo studies showed that both ENAH knockdown and daunorubicin treatment resulted in a favourable tumour regression in HCCLM3 CDX model (Figure 3J–O). PDX model with high ENAH amplification also showed marked tumour reduction upon daunorubicin treatment and no significant signs of weight loss at 1.25 mg/kg (Figure 3P–T).

In conclusion, this report highlights ENAH as a drug‐targetable protein identified through multi‐omics analyses, and uncovers a novel role in regulating ERK1/2/c‐Fos activity. Meanwhile, our results demonstrate that daunorubicin can directly target ENAH and serve as a powerful ENAH inhibitor for HCCLM3 treatment. Notably, our study delineates the multi‐omics landscape and molecular features that drive the HCC phenotypes, enabling a deeper understanding of the mechanisms underlying HCC progression and providing an exemplary demonstration for further precision‐targeted medicine research.

## AUTHOR CONTRIBUTIONS

Ming‐Gao Zhao, Wei‐Ming You and Zhi‐Mei Li conceived the project, designed the experiments, and wrote the paper. Zhi‐Mei Li performed the proteomics, transcriptomic, and bioinformatics analysis. Qing‐Qing Liu performed the molecular docking experiments. Zhi‐Mei Li and Guan Liu performed the pharmacological experiments. All authors critically revised the content and approved the final version for submission.

## FUNDING

This work was supported by the Natural Science Foundation of Shaanxi Province 2022JQ‐815.

## CONFLICT OF INTEREST STATEMENT

The authors declare no conflicts of interest.

## ETHICS STATEMENT

The HCC tissues and NAT samples used in this study were obtained from the Tangdu Hospital of Air Force Military Medical University with the approval of the Research Ethics Committee of the hospital. Written informed consent was obtained from all patients. All experimental and animal care protocols were approved by the Laboratory Animal Center of the Northwestern Polytechnical University and the Ethics Committee.

## Supporting information




**Supporting File 1**: ctm270366‐sup‐0001‐SuppMat.docx

**Supporting File 2**: ctm270366‐sup‐0002‐figureS1.png

**Supporting File 3**: ctm270366‐sup‐0003‐figureS2.png

**Supporting File 4**: ctm270366‐sup‐0004‐figureS3.png

**Supporting File 5**: ctm270366‐sup‐0005‐figureS4.png

**Supporting File 6**: ctm270366‐sup‐0006‐tableS1.docx

**Supporting File 7**: ctm270366‐sup‐0007‐tableS2.xlsx

**Supporting File 8**: ctm270366‐sup‐0008‐tableS3.xlsx

**Supporting File 9**: ctm270366‐sup‐0009‐tableS4.xlsx

**Supporting File 10**: ctm270366‐sup‐0010‐tableS5.xlsx

**Supporting File 11**: ctm270366‐sup‐0011‐tableS6.xlsx

**Supporting File 12**: ctm270366‐sup‐0012‐tableS7.xlsx

**Supporting File 13**: ctm270366‐sup‐0013‐tableS8.docx

**Supporting File 14**: ctm270366‐sup‐0014‐tableS9.docx

## Data Availability

All data needed to evaluate the conclusions in the paper are present in the paper and/or the Supplementary Material. Additional data related to this paper may be requested from the authors.

## References

[1] Deng G , Luo Y , Zhang Y , et al. Enabled homolog (ENAH) regulated by RNA binding protein splicing factor 3b subunit 4 (SF3B4) exacerbates the proliferation, invasion and migration of hepatocellular carcinoma cells via Notch signaling pathway. Bioengineered. 2022;13:2194‐2206. 35030977 10.1080/21655979.2021.2023983 PMC8973836

[2] Opitz R , Muller M , Reuter C , et al. A modular toolkit to inhibit proline‐rich motif‐mediated protein‐protein interactions. Proc Natl Acad Sci U S A. 2015;112:5011‐5016. 25848013 10.1073/pnas.1422054112 PMC4413326

[3] Gui J , Zhou H , Wan H , et al. The role of vasodilator‐stimulated phosphoproteins in the development of malignant tumors. Curr Cancer Drug Targets. 2024;24:477‐489. 37962042 10.2174/0115680096262439231023110106 PMC11092557

[4] Melchionna R , Spada S , Di Modugno F , et al. The actin modulator hMENA regulates GAS6‐AXL axis and pro‐tumor cancer/stromal cell cooperation. EMBO Rep. 2020;21:e50078. 32909687 10.15252/embr.202050078 PMC7645265

[5] Ito Y , Sasaki Y , Horimoto M , et al. Activation of mitogen‐activated protein kinases/extracellular signal‐regulated kinases in human hepatocellular carcinoma. Hepatology. 1998;27:951‐958. 9537433 10.1002/hep.510270409

[6] Zhang H , Xu Z , Xu Z , et al. The development of alpha, beta‐unsaturated lactam‐based andrographolide derivatives as anti‐gastric cancer agents with the ability of inhibiting the ERK/c‐Fos/Jun pathway. Eur J Med Chem. 2025;286:117291. 39848034 10.1016/j.ejmech.2025.117291

[7] Di Modugno F , DeMonte L , Balsamo M , et al. Molecular cloning of hMena (ENAH) and its splice variant hMena+11a: epidermal growth factor increases their expression and stimulates hMena+11a phosphorylation in breast cancer cell lines. Cancer Res. 2007;67:2657‐2665. 17363586 10.1158/0008-5472.CAN-06-1997 PMC3156572

[8] Han C , He S , Wang R , et al. The role of ARHGAP9: clinical implication and potential function in acute myeloid leukemia. J Transl Med. 2021;19:65. 33579308 10.1186/s12967-021-02733-5 PMC7881617

