# Supplementary material for: Targeting enabled homolog with daunorubicin inhibits ERK1/2/c‐Fos pathway and suppresses hepatocellular carcinoma progression
Source: Clin Transl Med. 2025 Jun 9;15(6):e70366. doi: 10.1002/ctm2.70366 (PMC12148948; doi:10.1002/ctm2.70366)
Supplement: Supplementary file 1 — Supporting File 1: ctm270366‐sup‐0001‐SuppMat.docx [file CTM2-15-e70366-s007.docx]

**Supplementary Materials**

**Methods**

**Sample Selection and Preparation**

The HCC and normal paired adjacent tissue (NAT) samples used in this study were obtained from the Tangdu Hospital of Air Force Millitary Medical University with the approval of the Research Ethics Committee of the hospital. Written informed consent was obtained from all patients.

Surgically resected paired tissues were selected from 20 patients with early-stage HCC (Barcelona Clinic Liver Cancer stage 0/A) who had not previously undergone radiotherapy or chemotherapy. All cases had HBV-infection background and were negative for lymphatic metastasis. Multi-omics profiling focused on HBV^+^/HCV^-^ male patients aged 40-60 years with BCLC-A HCC to standardize virological, demographic, and staging variables. Therefore, four cases (L03, L04, L05, and L07) were selected out. Clinical information, including HBV/HCV-infection background, age, gender, Barcelona Clinic Liver Cancer stage, tumor number, tumor diameter, and microvascular invasion status, was collected (Table S1). After surgical resection, the samples for proteomic analysis were frozen in liquid nitrogen for storage before use. The tissue samples for mRNA-seq were placed in RNAlater and stored at -80 °C. The formalin-treated tissues were used for immunohistochemistry evaluation.

**Proteomic Data Acquisition and Analysis**

Minced liver tissues were lysed in a lysis buffer containing protease and phosphatase inhibitors, followed by 1.5 min of sonication. The lysate was centrifuged, and the supernatant was collected as the whole-tissue extract. The protein concentration was measured using BCA assay. The sample concentration was adjusted to 1 mg/mL using lysis buffer, and the protein solution was digested with trypsin.

Peptides from the samples were labeled with TMT reagents. The TMT reagents were dissolved in anhydrous acetonitrile, and 15 μL of each TMT reagent was added to the corresponding aliquot of peptides. The reaction was incubated for 1 h with shaking and quenched with 2 μL hydroxylamine (5%) for 15 min, followed by desalting through C18 cartridges and vacuum-drying using Speed Vac.

Tryptic peptides were fractionated by high-pH HPLC to decrease sample complexity. Briefly, the tryptic peptides were dissolved in buffer A (10 mM ammonium acetate, pH 10). Then, they were loaded onto an Xbridge BEH C18 XP Column and eluted with a 60 min gradient from 5 to 90% of buffer B (10 mM ammonium acetate, 10% H_2_O, 90% ACN, pH 10).

The peptide samples were analyzed using an EASY-nLC 1200 LC system coupled to a Q Exactive HFX Orbitrap instrument (Thermo Fisher Scientific) with a nano-electrospray ion source. Peptides were re-dissolved in mobile phase A (0.1% formic acid and 2% ACN) and directly loaded onto a 15 cm long, home-made C18 nano-capillary analytical column (100 μm inner diameter) packed with 1.9 μm Reprosil-Pur C18-AQ beads. The peptides were separated with a 90 min gradient (phase A: 0.1% formic acid in water and 2% ACN; phase B: 0.1% formic acid in 80% ACN) at a constant flow rate of 300 nL/min (0-70 min, 2 to 22% of buffer B; 70-88 min, 22% to 95% of buffer B; 88-90 min, 95% of buffer B). Mass spectrometry was performed in the data-dependent acquisition (DDA) mode. For the MS1 full scan, ions with an m/z range of 350 to 1600 were acquired using an Orbitrap analyzer at a resolution of 120,000. For MS2, the resolution was set to 45k with a fixed first mass of 110 m/z. The automatic gain control (AGC) target was set as 3E6 with a maximal ion injection time of 30 ms, and 1E5 for MS2 with a maximal ion injection time of 96 ms. Precursor ions were selected and fragmented by higher energy collision dissociation (HCD) with a normalized collision energy (NCE) of 32%. The dynamic exclusion time was 45 s, and single-charged peaks as well as peaks with charges exceeding 6 were excluded from the DDA procedure.

Raw MS files generated by LC-MS/MS were processed against the UniProt human proteomic databases using MaxQuant (version 1.6.5.0) software on a Linux OS server enabled by the Andromeda search engine. Carbamidomethyl (C) was considered as a fixed modification, oxidation (M) and acetylation (Protein N-term) were used as variable modifications. The cutoff of the false discovery rate (FDR) using a target/decoy strategy was set to 0.01 for both proteins and peptides. The protease used was Trypsin/P. Up to two missed cleavage(s) was permitted. Peptide identification was performed using the Andromeda software. Finally, all the peptides and proteins matching the reversed database were filtered.

The density plots of the normalized intensities of the identified proteins showed that four paired samples passed the quality control and conformed to an expected unimodal distribution.

**mRNA-seq Data Acquisition and Analysis**

RNA was extracted from tissue samples by using the TRIzol reagent kit (Ambion, Invitrogen, USA) according to the manufacturer’s protocols. Sequencing libraries were generated using the TruSeq RNA LT Sample Prep Kit for Illumina following the manufacturer’s recommendations. Briefly, mRNA was purified using poly-T oligo-attached magnetic beads. After fragmentation, the first-strand cDNA was synthesized using RNase H and random hexamer primers. The second-strand cDNA was synthesized subsequently using dNTPs, buffer, RNase H, and DNA polymerase I. The library fragments were purified using QiaQuick PCR kits, followed by end repair, A-tailing, and adaptor addition. The library was constructed after DNA fragments enrichment. The RNA concentration of the library was determined using the Quant-iT^TM^ PicoGreen dsDNA Assay Kit on Qubit. Samples were clustered using the cBot cluster generation system. After cluster generation, the libraries were sequenced on an Illumina HiSeq 6000 platform.

The mRNA-seq reads were adaptor-trimmed, and data quality was assessed using the FastQC software. The mapped reads were assembled into transcripts using the StringTie software. For quantification purpose, the relative abundance of the transcripts was assessed using a normalized metric, the Fragments Per Kilobase of transcript per Million mapped reads (FPKM).

**Differential Expression Analysis**

The simple linear model and moderated t-statistics were used to identify differentially expressed proteins/genes between tumors and matched NATs using the R/Bioconductor package limma. Differentially expressed molecules were defined as the significance threshold P < 0.05 and fold change (expressed as |log2(ratio of protein/mRNA abundance between tumor and NAT samples)| > 0.585) in this comparison.

**Enrichment Analysis**

Gene set enrichment analysis (GSEA) was performed for pathway enrichment analysis. GSEA evaluates whether a priori-defined sets of genes exhibit statistically significant cumulative changes in genes expression associated with a specific phenotype. An FDR value of 0.05 was set as the cutoff. The enrichment score in was calculated by first ranking the molecules from most to least significant with respect to the different phenotypes, and the entire ranked list was subsequently used to assess how the proteins in each gene set were distributed across the ranked list.

The differentially expressed proteins/genes were subjected to further enrichment analysis using the R/Bioconductor package clusterprofiler, including Gene Ontology (GO) and Kyoto Encyclopedia of Genes and Genomes (KEGG). The selected enrichment is shown in Supporting Information Fig.S1E-S1G.

**Survival Analysis**

The Kaplan-Meier survival curves and log-rank test were used to assess the overall survival (OS) of patients with different proteins expression levels in TCGA database. To evaluate the prognostic power of ENAH, we performed univariable and multivariable Cox regression analyses of clinical variables relevant to the progression of HCC. All statistical analyses were performed using R, and a significance value of 0.05 was used.

**IHC Staining and Analysis**

Formalin-fixed paraffin-embedded specimens were deparaffinized in xylene and hydrated using a graded alcohol series. Antigen retrieval and endogenous peroxidase activities were blocked with 3% H2O2 for 20 min. After blocking with bovine serum albumin (BSA) for 1 hour at room temperature, the specimens were incubated with the primary antibody at 4 °C overnight and then with the secondary antibody for 15 min. The slides were stained using 3, 3’-diaminobenzidine, and the cell nucleus was counterstained with hematoxylin. The ENAH antibodies used for IHC were diluted at a ratio of 1:500. Slide images were processed using Aipathwell software, and the mean density of positive staining was calculated as integrated optical density (IOD)/area.

**Cell Culture**

The human HCC cell lines Huh7, HCCLM3, and HepG2 were routinely maintained in Dulbecco’s modified Eagle’s medium (DMEM), and Bel7402 cells were grown in RPMI 1640. The media used contained 10% fetal bovine serum (FBS; Invitrogen) and 1% penicillin-streptomycin solution (Invitrogen). All cells were incubated under a humidified atmosphere containing at 37 °C with 5% CO_2_.

**Lentivirus-mediated Knockdown of ENAH Expression**

The ENAH shRNAs and LV3 (H1/GFP&Puro) vectors were obtained from GenePharma (Shanghai, China). Control lentiviruses and lentiviruses encoding shRNAs targeting ENAH were generated according to the manufacturer’s instructions. After HCCLM3 cells were infected with lentiviruses for 24 h, stable cell lines were obtained for the following experiments by puromycin selection (2 μg/mL) for 3 days.

The ENAH shRNA sequences are as follows:

| No. | Sequence |
| --- | --- |
| shENAH-1 | GCAGCAAAGAGGATGCCAATG |
| shENAH-3 | GCAAGAAAGGGAGCGACAAGA |
| shENAH-2 | GGTTGGAGAGAGAGAGGTTAG |

**Plasmids, SiRNA, and Transfection**

ENAH pcDNA3.1 (ENAH-OE), ARHGAP9 pcDNA3.1 (ARHGAP9-OE), and empty vectors were subcloned into the expression vector pcDNA3.1 (Invitrogen, USA). Small-interfering RNA of ENAH or ARHGAP9 (si-ENAH and si-ARHGAP9, respectively), and scramble siRNA (si-NC) were purchased from GenePharma (Shanghai, China). The cells were seeded into six-well plates and cultured overnight until a 70–80% confluence. Transfection was performed using Lipofectamine 2000 (Invitrogen, Carlsbad, CA, USA) according to the manufacturer’s instructions.

**Western Blot Analysis**

The supernatants and intact cells were lysed with a loading buffer (50 mM Tris-HCl (pH 7.0), 100 mM dithiothreitol, 10% glycerol, 2% SDS, and 0.1% bromophenol blue) containing 1% protease inhibitor and 1% phosphatase inhibitor. The cell extracts were prepared for western blot. The protein concentration was quantified using a BCA kit. Equalized amounts of protein in each sample were loaded onto SDS-PAGE gels and transferred onto PVDF membranes. After blocking with 5% non-fat dry milk for 2 h, the membranes were incubated with the primary antibody for 1 h at room temperature, followed by incubation with horseradish peroxidase-conjugated secondary antibodies. The protein bands were visualized using the ECL chemiluminescence detection kit.

**Molecular Docking**

Molecular docking was performed using Discovery Studio 2021. The chemical structures of the compounds were inputted as mol2 files for docking. The ENAH crystal structure was obtained from the Protein Data Bank (<http://rcsb.org/>). Before docking, the ENAH protein structure was prepared by Protein Preparation, including the addition of hydrogen atoms, removal of unnecessary water molecules, and minimization. The binding site for molecular screening was defined based on the current ligand of ENAH using the Receptor-Ligand Pharmacophore Generation module. Then, the substrate was extracted from the protein for further screening and docking. The active compounds were ranked based on their -CDOCKER energies, hydrogen bond interactions, and binding mode patterns. All 3D diagrams of molecule-protein interactions were generated using PyMol 3.7.

**Cell Viability Assay**

Cells were seeded in 96-well plates (5000 cells/well) with different doses of compounds and incubated for 24 h. This was followed by the addition of an FBS-free medium containing 10% CCK-8 to each well and incubation for 1 h at 37 °C. The absorbance was measured at 450 nm using an enzyme-linked immunosorbent assay reader. The IC_50_ was calculated using the GraphPad Prism software.

**Colony Formation Assay**

HCCLM3 cells were treated with different doses of compound **11** (dissolved in H_2_O to make a stock solution, aliquoted, and stored at -20 °C.) for 14 days. Colonies were fixed and stained with 0.5% crystal violet. The colony numbers were counted for analysis.

**EdU Proliferation Assay**

HCCLM3 cells were plated in 24-well plates (5 × 10^4^ cells/well) for 24 h. Then, they were incubated with serum-free DMEM containing 50 μM EdU (RiboBio, Guangzhou, China) for 2 h, and treated with different doses of compound **11** or H_2_O (negative control) for 6 h. The cells were fixed for 30 min using 4% polyformaldehyde. This was followed by Apollo staining and DNA staining according to the manufacturer’s instructions. Cell images were captured using a fluorescence microscope.

**Cell Apoptosis Analysis**

Apoptosis induction was performed by double staining with Annexin V and propidium iodide (PI). After seeding in 6-well plates (270 × 10^3^ cells/well) and growing for 48 h, the HCCLM3 cells were treated with different doses of compound **11** for 24 h, followed by incubation with Annexin V-FITC and 5 mg/mL PI in the dark. The apoptotic cells were analyzed using a FACSCalibur flow cytometer.

**Wound Healing Assay**

Cells were seeded in 6-well plates and incubated till 90% confluence. The monolayers were scratched in straight lines with a 200-μL pipette tip. After washing with PBS, the cells were incubated in a serum-free medium containing different doses of compound **11**. Images were captured under an inverted microscope and analyzed using ImageJ software.

**Cell Migration Assay**

For the transwell migration assay, serum-starved cells were trypsinized and plated to the FN-coated upper chamber membrane with 8-μm pore filter (Corning Costar) of a transwell chamber with the corresponding dose of compound **11**. The lower transwell chamber was filled with complete medium. After incubation for 24 h, the cells on the membrane were fixed with methanol, and the migrated cells under the membrane were stained with crystal violet (0.5%). The dye was washed off with water, and the cells were examined under a microscope.

**Cell Cycle Analysis**

HCCLM3 cells from different groups were harvested, then washed with PBS, and fixed in ethanol (75%) for 2 h. After staining with the PI/RNase buffer in the dark for 15 min, the cell cycle profiles were determined using flow cytometry.

**CETSA**

CETSA, based on ligand-induced stabilization of target proteins, was performed to confirm the interaction between ENAH and compound **11** *in vitro* using western blot assay. Briefly, HCCLM3 cells cultured with 90% confluence were treated with media containing H2O (negative control) or compound **11** (1 µM) for 12 h. After treatment, the cells were isolated by trypsin digestion, collected by centrifugation, and resuspended in PBS. The suspension was divided equally into 9 PCR tubes and heated at a gradient temperature from 37 °C to 61 °C for 3 min. Subsequently, the cells were analyzed by western blot assay.

**Co-IP Assay**

Cells were lysed in a RIPA buffer containing protease and phosphatase inhibitors for 30 min on ice. Next, the supernatants of cell extracts were collected and incubated with antibodies at 4°C for 2 h. This was followed by mixing with protein A/G agarose beads at 4 °C overnight on a rotating wheel. The beads were washed five times with IP buffer (10 mM Tris-HCl (pH 7.5), 1 mM EDTA, 1% Triton-X, 150 mM NaCl, 0.2 mM sodium orthovanadate) and subjected to western blot, which was described previously.

**The Study Conducted on Mice**

The animals were maintained in accordance with the Regulations of Experimental Animal Administration issued by the State Committee of Science and Technology of the People’s Republic of China. All experimental and animal care protocols were approved by the Laboratory Animal Center of the Northwestern Polytechnical University and the Ethics Committee.

**The HCCLM3 cell xenograft mouse models**: Five-to-six-week-old female BALB/c nude mice with an average body weight of 20-23 g, were housed under specific pathogen-free conditions according to the guidelines established by the Research Animal Resources, Laboratory Animal Center, The Fourth Military Medical University (China). Every mouse was inoculated subcutaneously (s.c.) into the left flank with 0.1 mL of a solution containing 1.65 × 10^6^ of the indicated HCCLM3-GFP or ENAH^low^ HCCLM3-GFP cells. After the development of palpable tumors, the mice were divided into four groups (n = 8 per group): (1) HCCLM3-GFP + H2O (intraperitoneal injection); (2) ENAH^low^ HCCLM3-GFP; (3) HCCLM3-GFP + 0.5 mg/kg daunorubicin (intraperitoneal injection); and (4) HCCLM3-GFP + 1.0 mg/kg daunorubicin (intraperitoneal injection). The tumor size was measured every 2 days using a caliper. Tumor volume was calculated using the formula: 0.5 × length × (width^2^). In vivo images were captured using a Fusion FX SPECTRA system. The mice were sacrificed by pentobarbital overdose after 20 days. Then the tumors were harvested, and tumor weights were recorded.

**The PDX model of HCC**: The axillary transplantation of xenograft tissue from a patient with HCC was conducted in NOD/SCID mice, when the tumor tissues grew to 0.7 cm^3^, we harvested the tissues and transplanted them into the axilla of BALB/c nude mice at 5 weeks old as subsequent generations. The mice were randomly assigned to control (PBS), HSA, HAS-daunorubicin (1.25 mg/kg, intraperitoneal injection every 2 days) groups. The tumor volumes were recorded every day. After 10 days of administration, the mice were sacrificed, and all the tumors were extracted and weighed.

**Statistical Analysis**

Differentially expressed molecules were defined as the significance threshold P < 0.05 and fold change (expressed as |log2(ratio of protein/mRNA abundance between tumor and NAT samples)| > 0.585) in this comparison. An FDR of 0.05 was set as the cutoff in the GSEA. Survival analyses were performed using R, and a significance value of 0.05 was used. The survival curves were calculated using the Kaplan-Meier method, and the differences were assessed through a log-rank test. Univariate and multivariate Cox regression analyses was used to determine the independent factors that influenced survival. In addition, the statistical significance of differences between groups in the *in vitro* and *in vivo* studies was assessed using an unpaired Student’s t test. The analyses were performed using GraphPad Prism 8.0 Software. Pearson’s correlation analysis was conducted to evaluated the association between genes and proteins. Results were considered statistically significant at P < 0.05. P values are displayed using a single asterisk for significances ranging from 0.05 to 0.01, two asterisks for values between 0.001 and 0.01, and three asterisks for values below 0.001.

**Figure legends**

**Figure S1** Muti-omics landscape of the HCC samples. (A-B) Overview of the differentially expressed proteins (A) and genes (B) between four tumors and paired NATs. (C-D) Consensus clustering based on the dysregulated proteins (top, C) or genes (D) between tumors and NATs among the proteomic or transcriptomic profiles. Each column represents a patient sample, and the rows indicate proteins. (E-F) Enriched pathways revealed by GSEA in HCC from our proteomic (E) or transcriptomic (F) study. (G) GO enrichment analysis for significantly differentially expressed proteins and mRNA associated with the HCC and NAT paired tissues. (H) Proteomic and transcriptomic analysis of the significantly differentially regulated pathways between tumors and NATs.

**Figure S2** Identification of ENAH as tumor-associated protein by multi-omics analyses. (A) Global heatmap of the selected overlap genes in (Figure 1A). The biological functions related to these genes are denoted on the right. (B) Correlation matrix of proteins/mRNA (in (A)) abundance among the proteomic/transcriptomic profiles. (C) Differential expression analysis of protein-mRNA correlated genes (in (B)) to identify the consistent upregulated (red) and downregulated (blue) genes in HCC from TCGA cohort. (D) The association of ENAH expression with four variables including HCC patients’ age, event, gender, and tumor stage, derived from TCGA cohort. (E) Expression profiles of ENAH in NATs, tumors, and HCC stages. (F) Expression profiles of ENAH in NATs and HCC grades. (G) Kaplan-Meier curves for the 5-year overall survival based on ENAH protein abundance in HCC tumors in the TCGA cohort. The P value was calculated using log-rank test. (H) IHC images of 20 HCC samples from our cohort showing the ENAH abundance in the tumor and NAT samples. The right panel shows the representative ENAH expression in the HCC tissues and paired NATs. (I) Comparison of the staining mean density with the ENAH antibodies between tumors and NATs. (J) Comparison of the ENAH abundance between tumor and normal tissues in various cancers from TCGA cohort. The P value was calculated using the Wilcoxon test. Significance levels are indicated as ^*^P＜0.05; ^**^P＜0.01; ^***^P＜0.001.

**Figure S3** Identification of the ENAH inhibitor using virtual ligand docking. (A) Structure of compounds **1**-**14**. (B-D) Binding conformations of daunorubicin bound to ENAH generated by molecule docking. (B) Daunorubicin was observed to occupy the binding pocket in ENAH (PDB: 6RCF). (C) Alignment of daunorubicin (yellow) and the ENAH original ligand (blue) at the binding site of the ENAH. (D) Illustration of the interactions between daunorubicin and ENAH. The hydrogen bond is shown as a green line, and the hydrophobic interaction is shown as a purple line.

**Figure S4** The inhibition of ENAH with daunorubicin effectively suppresses the ERK1/2/c-Fos signaling pathway. (A) Co-expression analysis to identify the significant ENAH-associated genes in TCGA cohort. (B) The top positively and negatively co-expressed genes of ENAH in HCC. (C) The structure scheme of ARHGAP9. (D) Network diagram depicting the relationship between ARHGAP9 and the ERK1/2-related pathway.
