# Supplementary figures and images for: Targeting enabled homolog with daunorubicin inhibits ERK1/2/c‐Fos pathway and suppresses hepatocellular carcinoma progression
Source: Clin Transl Med. 2025 Jun 9;15(6):e70366. doi: 10.1002/ctm2.70366 (PMC12148948; doi:10.1002/ctm2.70366)

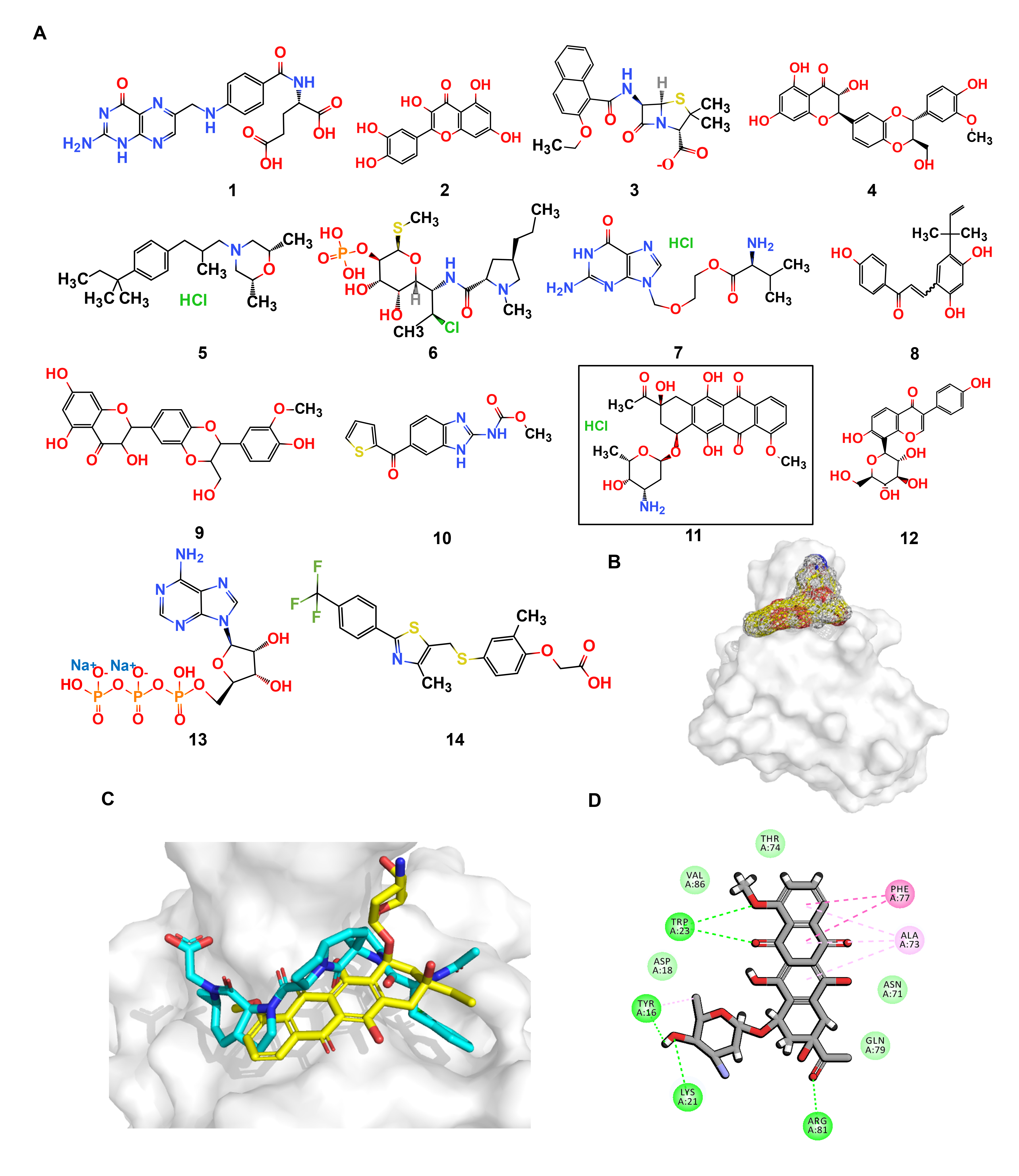

Supplement: Supplementary file 4 — Supporting File 4: ctm270366‐sup‐0004‐figureS3.png [file CTM2-15-e70366-s009.png]

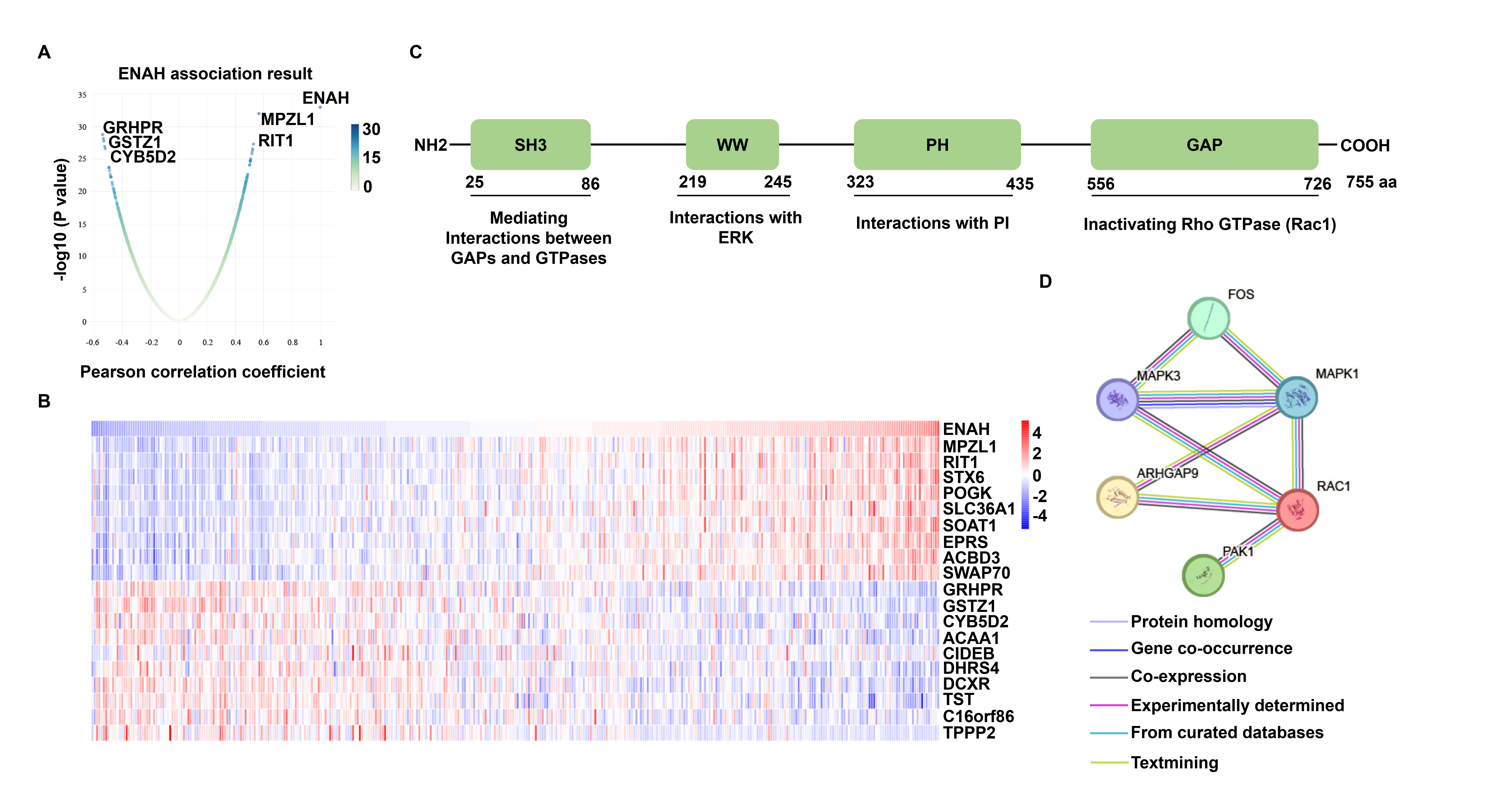

Supplement: Supplementary file 5 — Supporting File 5: ctm270366‐sup‐0005‐figureS4.png [file CTM2-15-e70366-s014.png]
