# Supplementary material for: Targeting enabled homolog with daunorubicin inhibits ERK1/2/c‐Fos pathway and suppresses hepatocellular carcinoma progression
Source: Clin Transl Med. 2025 Jun 9;15(6):e70366. doi: 10.1002/ctm2.70366 (PMC12148948; doi:10.1002/ctm2.70366)
Supplement: Supplementary file 6 — Supporting File 6: ctm270366‐sup‐0006‐tableS1.docx [file CTM2-15-e70366-s003.docx]

**Table S1.** Detailed clinical information of 20 cases.

| Case No. | Gender | Age | HBV^a^ | HCV^a^ | BCLC stage | Tumor number | Diameter of tumor (cm) | Microvascular invasion^b^ | Lymphatic metastasis^b^ | Cancer recurrence^b^ | Proteomic^b^ | mRNA-Seq^b^ |
| --- | --- | --- | --- | --- | --- | --- | --- | --- | --- | --- | --- | --- |
| L01 | M | 53 | P | P | A | 1 | 10.2 | 0 | 0 | 0 | 0 | 0 |
| L02 | M | 49 | P | P | A | 1 | 1.2 | 0 | 0 | 0 | 0 | 0 |
| L03 | M | 53 | P | N | A | 1 | 7.3 | 0 | 0 | 0 | 1 | 1 |
| L04 | M | 44 | P | N | A | 1 | 8 | 0 | 0 | 0 | 1 | 1 |
| L05 | M | 57 | P | N | A | 1 | 1 | 0 | 0 | 0 | 1 | 1 |
| L06 | M | 36 | P | N | A | 1 | 5 | 0 | 0 | 0 | 0 | 0 |
| L07 | M | 52 | P | N | A | 1 | 3.5 | 0 | 0 | 0 | 1 | 1 |
| L08 | M | 70 | P | N | A | 1 | 3.3 | 1 | 0 | 0 | 0 | 0 |
| L09 | M | 63 | P | N | A | 1 | 2.2 | 0 | 0 | 0 | 0 | 0 |
| L10 | F | 62 | P | N | A | 1 | 4.1 | 0 | 0 | 0 | 0 | 0 |
| L11 | M | 25 | P | N | A | 1 | 1.6 | 1 | 0 | 0 | 0 | 0 |
| L12 | M | 47 | P | N | A | 1 | 8 | 1 | 0 | 0 | 0 | 0 |
| L13 | M | 68 | P | N | A | 1 | 5 | 0 | 0 | 0 | 0 | 0 |
| L14 | F | 41 | P | N | A | 1 | 1.5 | 0 | 0 | 0 | 0 | 0 |
| L15 | F | 62 | P | N | A | 1 | 4.1 | 0 | 0 | 0 | 0 | 0 |
| L16 | M | 53 | P | P | A | 1 | 1.8 | 0 | 0 | 0 | 0 | 0 |
| L17 | M | 56 | P | N | 0 | 1 | 1.5 | 0 | 0 | 0 | 0 | 0 |
| L18 | M | 52 | P | P | A | 1 | 5.7 | 0 | 0 | 0 | 0 | 0 |
| L19 | M | 47 | P | P | A | 1 | 1.5 | 0 | 0 | 0 | 0 | 0 |
| L20 | M | 17 | P | N | A | 1 | 7.5 | 0 | 0 | 0 | 0 | 0 |

a: N=Negative, P=Positive; b: 0=No, 1=Yes.
