# Supplementary material for: Targeting enabled homolog with daunorubicin inhibits ERK1/2/c‐Fos pathway and suppresses hepatocellular carcinoma progression
Source: Clin Transl Med. 2025 Jun 9;15(6):e70366. doi: 10.1002/ctm2.70366 (PMC12148948; doi:10.1002/ctm2.70366)
Supplement: Supplementary file 13 — Supporting File 13: ctm270366‐sup‐0013‐tableS8.docx [file CTM2-15-e70366-s002.docx]

**Table S8** Binding affinity of the screening drugs.

| No. | Names | -CDOCKER energy^a^ (kcal/mol) |
| --- | --- | --- |
| **1** | Folic acid | 51.3494 |
| **2** | Quercetin | 55.6988 |
| **3** | Nafcillin sodium salt monohydrate | 1.24048 |
| **4** | Silibinin | 48.1995 |
| **5** | Amorolfine hydrochloride | 17.4596 |
| **6** | Clindamycin phosphate | 33.2144 |
| **7** | Valacyclovir hydrochloride | 38.2342 |
| **8** | Licochalcone-A | 27.5527 |
| **9** | Silymarin | 42.6916 |
| **10** | Nocodazole | 17.1100 |
| **11** | Daunorubicin | 15.8223 |
| **12** | Puerarin | 33.2712 |
| **13** | ATP disodium salt | 41.0177 |
| **14** | GW-501516 | 33.0532 |

^a^ Docking score/interaction potential of compounds with ENAH (kcal/mol).
