# Supplementary material for: Targeting enabled homolog with daunorubicin inhibits ERK1/2/c‐Fos pathway and suppresses hepatocellular carcinoma progression
Source: Clin Transl Med. 2025 Jun 9;15(6):e70366. doi: 10.1002/ctm2.70366 (PMC12148948; doi:10.1002/ctm2.70366)
Supplement: Supplementary file 14 — Supporting File 14: ctm270366‐sup‐0014‐tableS9.docx [file CTM2-15-e70366-s013.docx]

**Table S9** IC_50_ value (μM) of compounds 1-14 against two HCC cell lines.

| No. | HCC cell lines | |
| --- | --- | --- |
|  | HCCLM3 | Bel7402 |
| **1** | > 200 | > 200 |
| **2** | > 200 | > 200 |
| **3** | > 200 | > 200 |
| **4** | > 200 | > 200 |
| **5** | > 200 | > 200 |
| **6** | > 200 | > 200 |
| **7** | > 200 | > 200 |
| **8** | > 200 | > 200 |
| **9** | > 200 | > 200 |
| **10** | > 200 | > 200 |
| **11** | 7.42 ± 0.06 | > 200 |
| **12** | > 200 | > 200 |
| **13** | > 200 | > 200 |
| **14** | > 200 | > 200 |
